# A novel approach for automated counting of tumor cell colonies

**DOI:** 10.1371/journal.pone.0354467

**Published:** 2026-07-23

**Authors:** Manuel G. Forero, Laura A. Medina, Andrés Felipe Patiño-Aldana, Andrea Del Pilar Hernandez-Rodríguez, Gabriela López-Molina, Harold H. Mena, Mateo Díaz-Quiroz, Margarita Garcia, Paulo Quintero, Juliana Sandoval-Navia, Alejandro Ondo-Méndez

**Affiliations:** 1 Faculty of Engineering, Lún Research Seedbed, Universidad de Ibagué, Ibagué, Colombia; 2 Clinical Research Group - Research Seedbed in Biochemistry, Cancer and Radiobiology, Universidad del Rosario, Bogotá, Colombia; 3 Faculty of Science and Engineer, University of Hull, Hull, United Kingdom; 4 Centro de Control de Cáncer Ltda, Bogotá, Colombia; University of Nebraska Medical Center, UNITED STATES OF AMERICA

## Abstract

**Background and Objective:**

The heterogeneous response of cancer cells to radiation necessitates comprehensive investigations into cell survival, which are often conducted using clonogenic assays. These assays involve laborious and subjective manual enumeration of cell colonies. This paper presents an innovative digital image processing technique for automated cell colony counting.

**Methods:**

This research focused on HT-29 cells (colon cancer). The methodology uses Hough techniques to achieve precise detection, mathematical morphology, and establishment of the optimal minimum colony size using ROC curve. This approach seamlessly integrated into ImageJ software as a ColCounter plugin.

**Results:**

Images with resolution of 1200*1200 pixels achieved sensitivity and specificity of 92.88% and 92.62%, respectively. To determine the software´s effectiveness of image analysis of different resolutions compared with manual counting, an evaluation of the automated method’s reliability was conducted. The findings revealed an overall intraclass correlation coefficient (ICC) of 0.89 (95% CI: 0.81–0.93). The limits of agreement were determined using the Bland-Altman method.

**Conclusions:**

The proposed methodology demonstrated interchangeability with the conventional manual enumeration technique. The results confirm the software’s elevated performance, even in altered contexts. ColCounter offers manual editing function, enhancing its utility as a streamlined, rapid, and precise tool for quantifying tumor cell colonies in clonogenic assays.

## 1. Introduction

Radiotherapy plays an important role in the treatment of cancer, being used in approximately 50% of patients [1]. Its antitumor effect is due to DNA damage through direct and indirect mechanisms, which activate cell death pathways [2]. However, DNA repair mechanisms may be activated in surviving cancer cells to maintain their proliferation capacity. In this context, clonogenic assays represent the *in vitro* gold standard for assessing radioresistance, as they evaluate the ability of cells to survive, proliferate, and form colonies after exposure to a cytotoxic agent [3–5]. Although widely used to study radioresistance, clonogenic assays are also employed in other oncologic fields like drug screening and quantifying **“**stemness**”** and tumorigenicity of cancer stem cells [6,7].

Cell survival curves describe the relationship between the radiation dosage and the survival cell fraction [8]. The construction of these graphics requires colony counting, which tends to be manual, making it a labor-intensive and error-susceptible procedure [9,10]. Automated and semi-automated colony count techniques based on image analysis have been proposed to enhance accuracy, reproducibility, and expedite the counting process. Examples of these techniques include ColonyCountJ, an ImageJ plugin for quantifying colony parameters in clonogenic assays [10]; CoCoNut, a standalone cell colony counter [11]; and a high-throughput method based on edge detection for automated colony and cell counting [12].

As automated methodologies are developed to tackle complex microscopy image analysis across diverse domains, conventional cell counting software often falls short when faced with colony counting challenges, which include automatically identifying wells, mitigating illumination inconsistencies inherent in scanner-based scans, and facilitating colony selection based on cell counts. Thus, an urgent need arises for techniques and software explicitly tailored to meet these requirements.

Research efforts in this particular domain keep on expanding, different proposals have been developed exploring distinct approaches like the use of the Watershed technique for image segmentation, estimation of well surface area covered by colonies, and the identification of regions of interest and local segmentation to identify and label cancerous colonies [11,13,14]. Some novel approaches rely on the area covered by the colony (ACC) rather than the colony count itself, using different techniques like spatial Fuzzy C-Means clustering, Circle Hough Transform, and local adaptive thresholding for image processing [15,16]. More recently, artificial intelligence-based models have been developed, for example, Klett et al. utilized a time-lapse microscopy for real-time tracking of colony formation, and Lavitt et al. developed a model based on convolutional neural networks for cell counting that could be extrapolated to clonogenic assays [17,18].

Although alternatives for colony counting have been designed, some limitations include high relative error values, restrictions when identifying merged or overlapping colonies, the need for high resolution images, calculation of the survival fraction based on the ACC, whose parameter, despite correlating with the count, does not replace it; and the requirement of specialized equipment for image acquisition that is difficult to access. It is still necessary to develop models that demonstrate good performance and that do not represent acquisition barriers.

Exploring different methods for developing tools that facilitate colony counting would lead to the generation of high-performance, user-friendly, and cost-effective techniques that overcome the limitations of manual counting. The present study aimed to describe the development of an innovative model based on digital image processing, specifically using the Hough technique, for automated cell colony counting “ColCounter” and to evaluate its performance compared with manual counting.

## 2. Materials and methods

### 2.1. Cell culture and irradiation protocols

**Fig 1:**
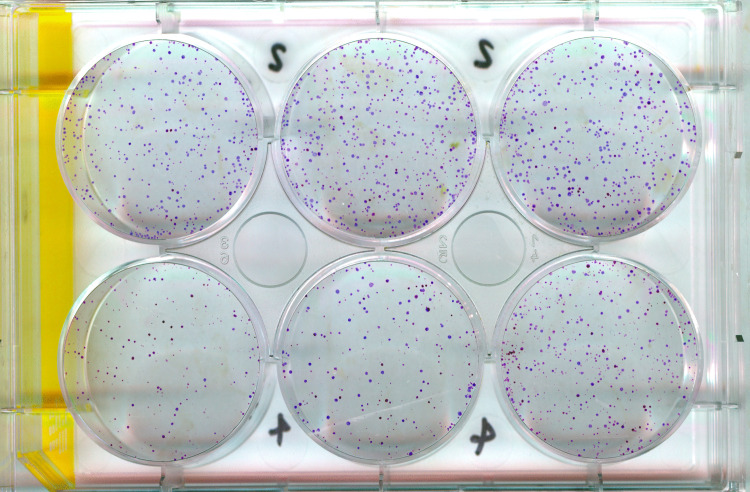
Image of a six well plate with violet stained colonies.

HT-29 cells (colorectal cancer; ATCC code HTB-38^TM^) [19] were grown in Dulbecco´s Modified Eagle´s medium (DMEM, Biowest®) supplemented with 10% Fetal Bovine Serum (FBS, Hyclone®) and seeded in 60 mm diameter cell culture dishes until they reached confluence. Cells were irradiated with 6 MV X-ray photons at doses of 0, 1, 2, 4, 6, 8, 10, 12, 16, and 20 Gy. To ensure electronic equilibrium and dose homogeneity in the target volume, the dishes were irradiated on a paraffin block. A clonogenic assay was performed following the plating after treatment protocol [3]. Cells were seeded in 6 well plates in triplicate in a range of 200–2000 cells per well, depending on the radiation dose. Cells were cultured in DMEM supplemented with 10% FBS and incubated at 37°C in an atmosphere containing 5% CO2 for 14 days to allow adhesion. Finally, colonies were fixed and stained with 4% formaldehyde and 1% crystal violet, respectively. The resulting images, encompassing six plates, were acquired using a Hewlett-Packard scanner (HP Scanjet Pro) at resolutions of 1024 x 5320 and 456 x 456 pixels, as illustrated in Fig 1.

**Fig 2:**
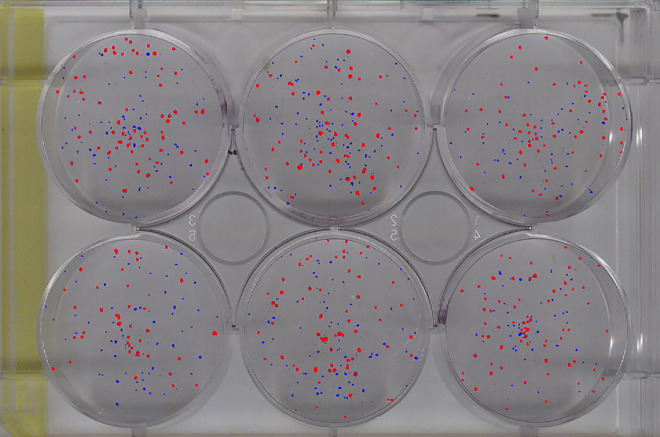
Ground-truth images for method validation. Red indicates colonies comprising 50 or more cells, and blue represents clusters of fewer than 50 cells that do not meet the criteria for classification as colonies.

In total, 17 six-well plates were used, resulting in a collective count of 102 wells, that were analyzed to determine the optimal color channel for subsequent processing. Among these, the colonies from 14 plates (equivalent to 84 wells) were identified manually by two specialists who collaborated to reach a consensus. This meticulous process yielded ground-truth images, exemplified in Fig 2, which were subsequently employed to define the minimum colony size, which is an essential parameter for developing the plugin. Next, 15 different plates were used to validate the method using manual counting as a comparative method. However, 4 wells out of these 15 plates (90 wells) were discarded due to poor scan quality (heterogenous light and dark-colored background). Finally, 86 wells were used for the concordance analysis.

### 2.2. Statistical analysis

To develop the plugin, a ROC curve was used to assess the sensitivity and false-positive rate (1-specificity). The objective of this approach was to determine the optimal minimum colony area that could achieve the highest true positive rate while simultaneously minimizing the false positive rate.

Once the plugin was configured, the concordance between manual count and plugin results was assessed. This evaluation aimed to gauge the plugin’s efficacy in scenarios where researchers are confronted with the necessity to work with images of resolutions other than those specified in the original protocol. To test the adaptability of the tool across various resolutions, photographs of different resolutions (200, 300, and 600 dpi) were used.

To quantify the level of agreement, the intra-class correlation coefficient was calculated. Furthermore, the Bland-Altman method was employed to investigate the average bias and delineate the limits of agreement between the two methods [20]. Additionally, a Passing-Bablok regression was fitted, enabling a non-parametric assessment of the null hypothesis concerning the equivalence of performance between manual counting and the ColCounter plugin [21]. To conduct statistical analysis, R software was employed in conjunction with packages including *tidyverse, irr, blandr,* and *mcr [22–26].*

### 2.3. Methods

**Fig 3:**
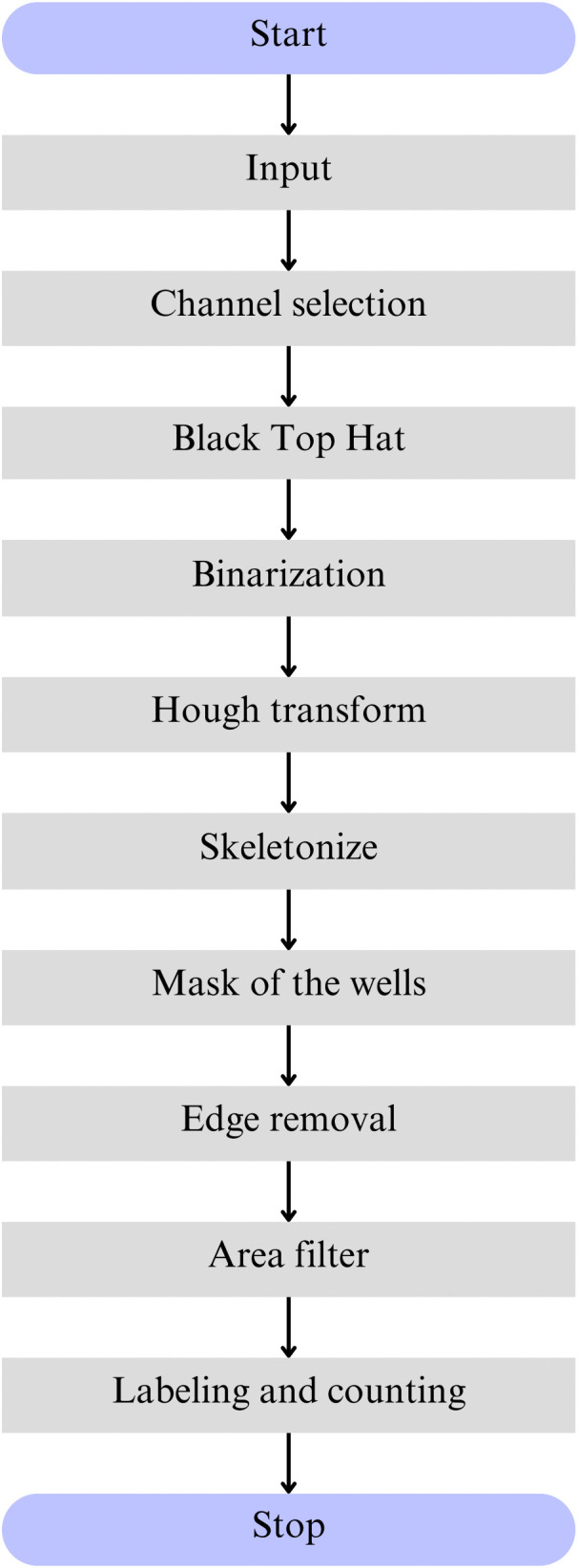
Illustration of the Developed Methodology’s Flowchart.

**Fig 4:**
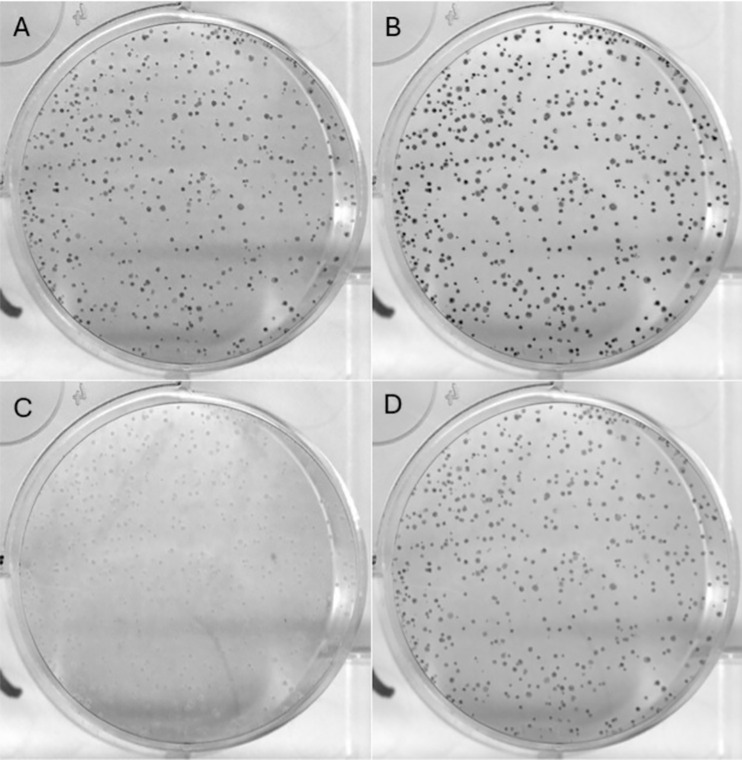
Images obtained from each color channel and monochrome images obtained by averaging channel values. A. Red channel. B. Green channel. C. Blue channel. D. Grayscale image.


Fig 3 shows a comprehensive flowchart of method development. Upon receiving the input image, the subsequent step involves selecting the color channel that provides the most informative data. It’s important to note that not all color channels offer relevant information. As depicted in Fig 1, colonies exhibit a faint purplish hue arising from the fusion of blue and red components, while the background maintains a grayish appearance. Consequently, the contrast between colonies and the lightly colored background in channels like red and blue was less pronounced compared to the green channel, due to the absence of green color within the colonies. The image representations for each color channel are shown in Fig 4. These images are accompanied by the resultant grayscale image generated by averaging the values from all three color channels. This computation is mathematically summarized in Equation 1.

$$\begin{array}{c} Grey(x,y)=\left(R(x,y)+G(x,y)+B(x,y)\right)/\text{3} \end{array}$$ (1)

To determine which color channel contained more information and mathematically verify the decision to select green for colony detection over the other two channels, the entropy—a measure of information—was calculated for each channel using Equation 2. Where *h* signifies the normalized histogram of each color channel, *q* represents the color level, and *L* pertains to the maximum intensity within each color channel. Table 1 lists the entropy values corresponding to each color channel and monochromatic image, obtained by averaging the color values from each channel across all pixels. The entropy values were measured across 17 plates. The outcomes demonstrate that the green channel consistently displayed the highest entropy values, which is consistent with the expectation. This outcome bolsters the rationale for selecting the green channel for subsequent processing of the entire set of images.

**Table 1:** Entropy metrics of image channels.

Image	Entropy of the channel			Entropy of the image in gray
	Red	Green	Blue	
1	13.2	13.02	13	12.9
2	13.24	13.31	13.29	13.24
3	13.17	13.22	13.18	13.13
4	13.17	13.22	13.18	13.14
5	13.13	13.16	13.15	13.13
6	12.87	12.94	12.85	12.85
7	13.06	13.11	13.06	13.02
8	12.93	12.97	12.92	12.91
9	13.13	13.16	13.13	13.11
10	13.48	13.54	13.5	13.47
11	13.28	13.35	13.3	13.3
12	13.02	13.04	13.03	13.02
13	12.99	13.02	13	12.98
14	13.11	13.15	13.12	13.13
15	15.53	15.76	15.49	15.56
16	15.24	15.52	15.41	15.4
17	15.13	15.33	15.32	15.2
Mean	13.5	13.577647	13.525294	13.499412

Entropy Values of Monochromatic and Color Channel Images across 17 Plates.


$$\begin{array}{c} \sum_{q=\text{0}}^{L-\text{1}}h(q){\text{log}}_{\text{2}}h(q) \end{array}$$ (2)

**Fig 5:**
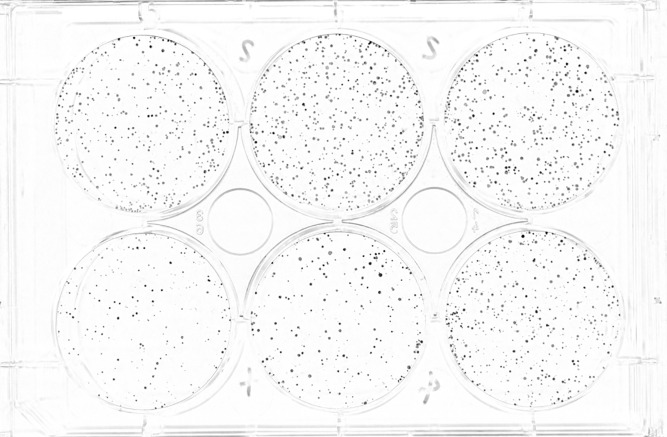
Resulting image of the Black Top Hat transformation.


Upon selecting the appropriate color channel, the images were segmented to distinguish colonies from background. However, because of the substantial variability in colony intensity within the images, a thresholding method was found unsuitable. Instead, Black Top Hat transformation was employed to distinguish between the background and darker regions, which corresponded to the colonies, as illustrated in Fig 5. This approach enhances the visibility of smaller, darker structures against a relatively uniform background. This was achieved by subtracting the morphological closing result from the original image. Mathematically, if *Q* represents the original image and *B* is the structuring element, the Black Top Hat transformation *T* is calculated as follows (Equation 3):

$$\begin{array}{c} T=Q-(Q\cdot B) \end{array}$$ (3)

In this study, an octagon with a radius larger than the measured colony size was chosen as the structuring element *B*. This transformation enhanced the contrast between the colonies and the background, facilitating the subsequent processing steps.

**Fig 6:**
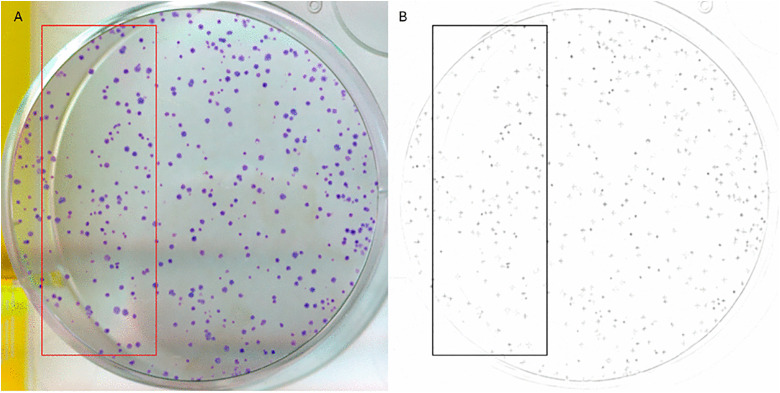
The Black Top Hat Operator eliminates artifacts caused by edge reflections on plates during scanning. A. Close-up view of a well extracted from the original image. The artifact resulting from the plate edge reflection on the well’s edge is highlighted within the red box. B. Result obtained by applying the Top Hat operator with an octagonal structuring element. The black box indicates the virtual disappearance of the artifact.

Another advantage of employing the Black Top Hat method instead of thresholding is its ability to address reflection artifacts that may arise during scanning. The shadow, with a radius larger than that of the colonies, was effectively removed, as demonstrated in Fig 6.

**Fig 7:**
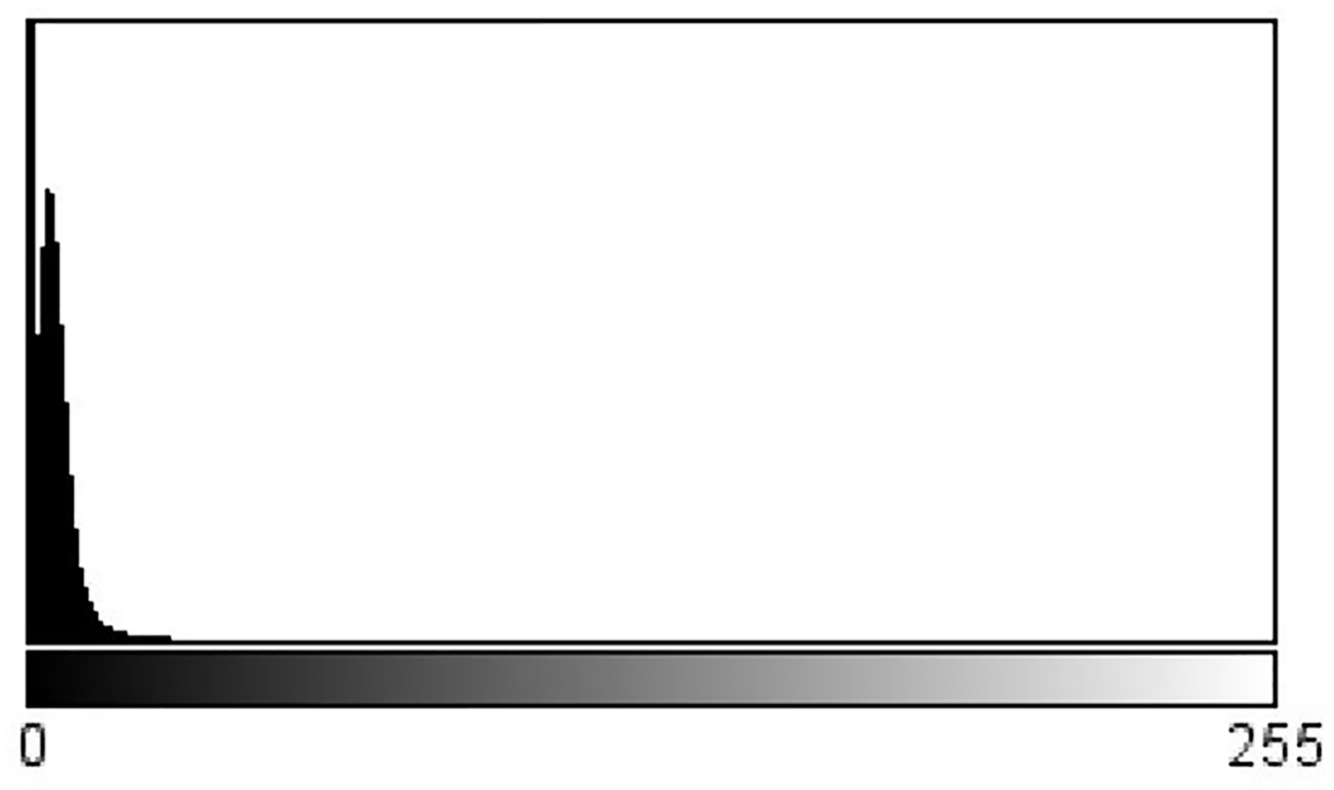
Typical histogram of a colony image after applying the Black Top Hat transformation.

**Fig 8:**
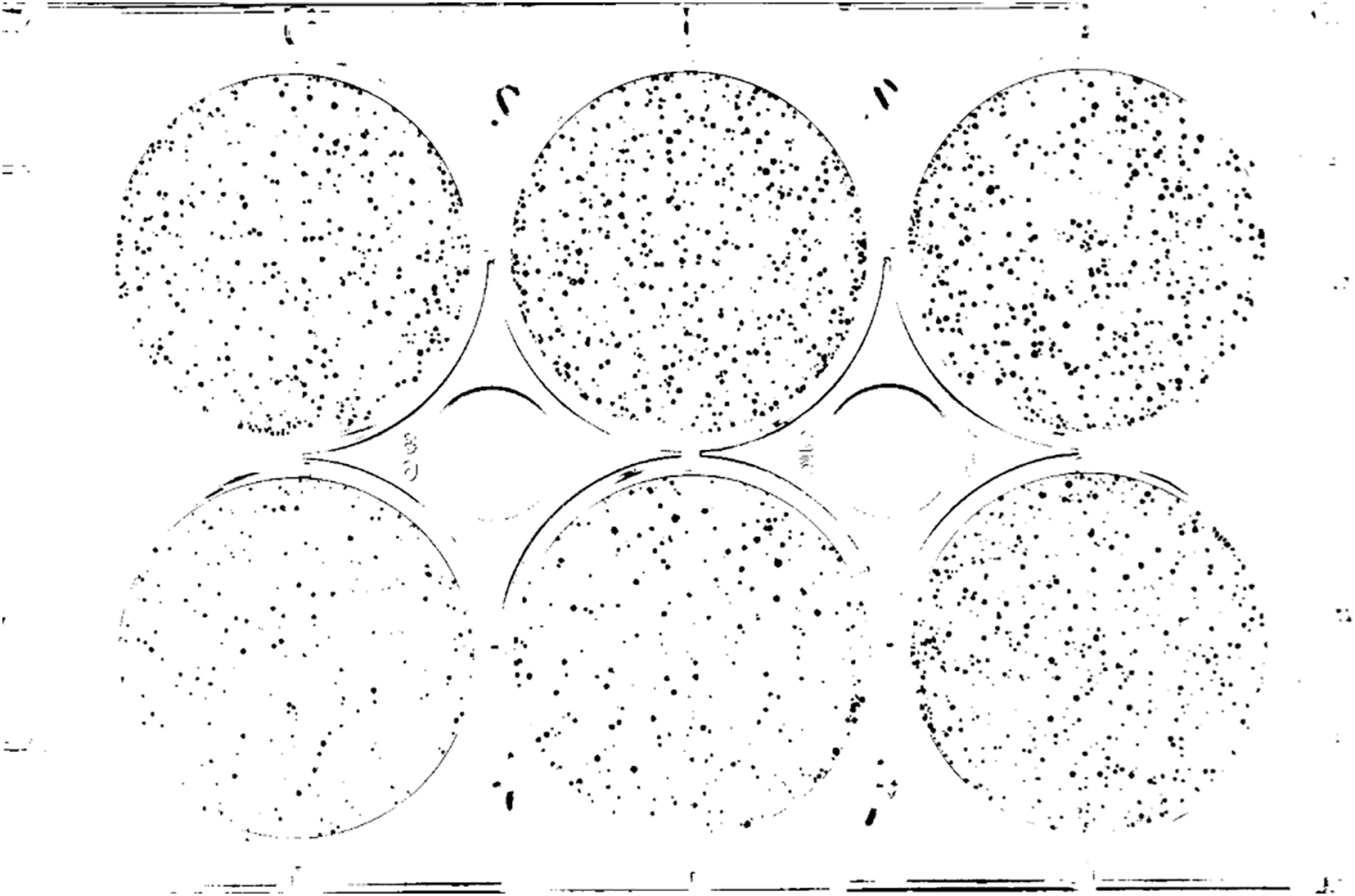
Image resulting from binarization using Otsu’s method of Fig 5.

To distinguish the colonies from the background, the Otsu thresholding method was employed, which yielded superior results compared with the other methods, despite the histogram not exhibiting two clearly identifiable Gaussian modes, as illustrated in Fig 7. Conversely, the histogram was predominantly unimodal, making it challenging to locate the mode corresponding to the colonies. Fig 8 shows the outcome of the thresholding process using the Otsu method.

**Fig 9:**
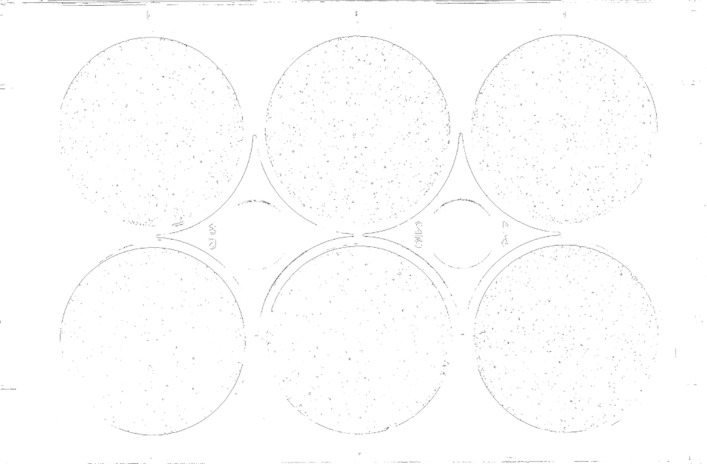
Application of skeletonization to the image in Fig 8. Reduction in white pixels enhances the well boundary localization efficiency.

To determine the number of colonies present, it is crucial to initially identify each well individually. Given the circular shape of the wells, two distinct techniques were assessed for detecting the edges of each plate. These methods include the Hough transform, which is commonly used to identify geometric shapes, and the Random Sample Consensus (RANSAC) technique. Both methods were optimized to specifically search for circles that closely matched the size of the wells. Furthermore, to mitigate the time complexity associated with assessing a significant number of pixels, the input binarized image underwent skeletonization, resulting in the reduction of the edges to a single-pixel width, as depicted in Fig 9. The Hough transform demonstrated superior performance for accurately identifying circles [27], thus establishing it as the preferred technique for this purpose.

**Fig 10:**
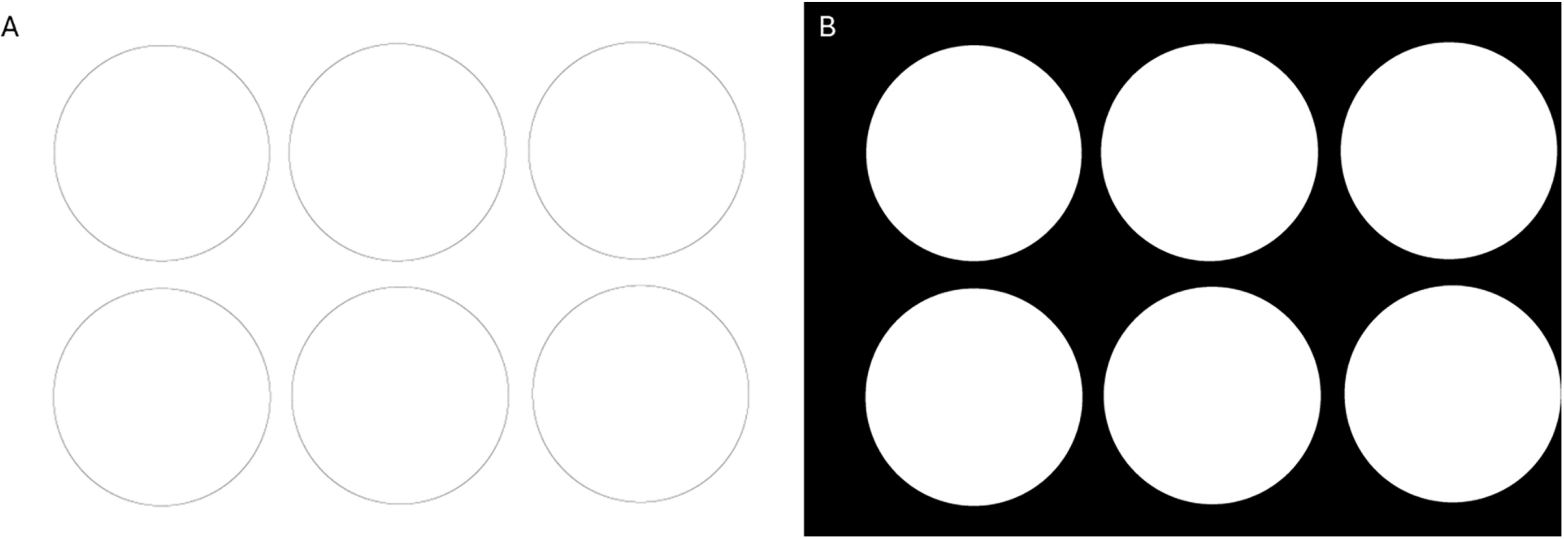
Locations of the circumferences correspond to each well detected by Hough’s method. A. Detected circumferences. B. Mask generated from image A using morphological filling.

**Fig 11:**
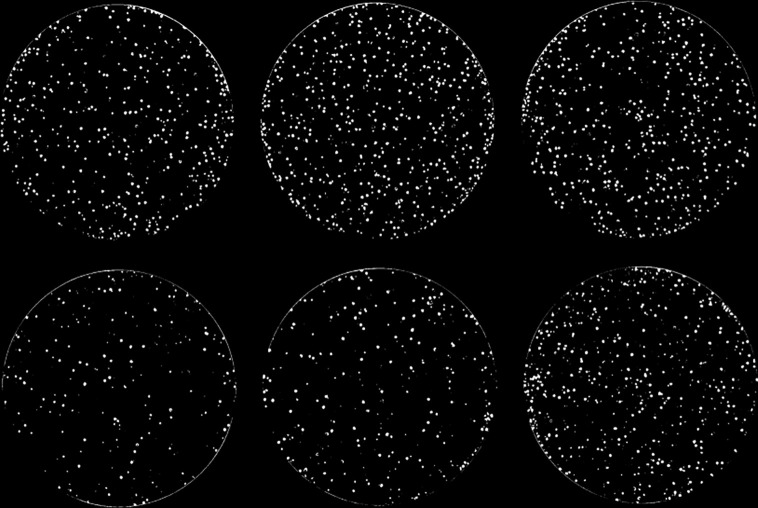
Image of colonies after background removal using the binary mask.

Following well localization, the resulting image was used to generate a mask using morphological filling, effectively separating the wells from the background, as shown in Fig 10. To isolate the colonies within all wells, the minimum value at each pixel between the binarized image, shown in Fig 8, and the mask was computed, as illustrated in Fig 11.

**Fig 12:**
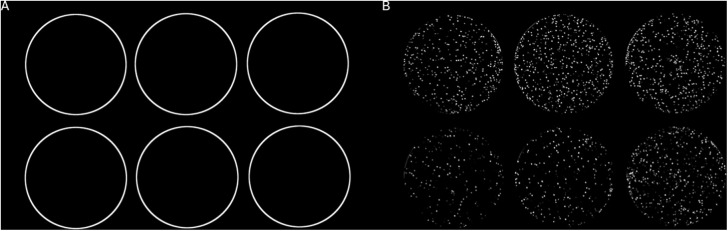
Removal of Plate Edges. A. Image of Dilated Circles. B. Image Resulting from Edge Removal.

Before colony counting, it is essential to eliminate the outline of each plate. To achieve this, after the colonies have been successfully separated from the background, the circular image obtained through the Hough transform is dilated and subtracted from the binarized image using Otsu’s method. The resulting output is shown in Fig 12.

### 2.4. Colony counting

**Fig 13:**
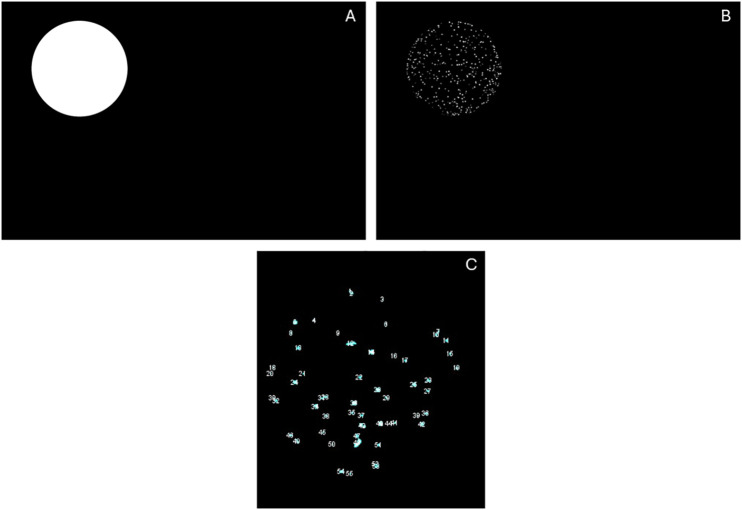
Automated identification and quantification of colonies within a well using the proposed method. A. Mask utilized to identify individual colonies in the plate. B. Colonies within the well. C. Labeled and counted colonies.

After segmenting the colonies and removing the background noise, each well was labeled using 8-connectivity. This assigns a distinct label to each well and creates a mask to identify each one of them. The objects detected within each well were then labeled, again using 8-connectivity, and subsequently, the colonies were counted, as demonstrated in Fig 13. The choice of 8-connectivity for labeling colonies rather than 4-connectivity is justified because the former is more suitable for fine object labeling, preventing situations in which colonies connected only by a diagonal pixel are marked as separate entities.

**Fig 14:**
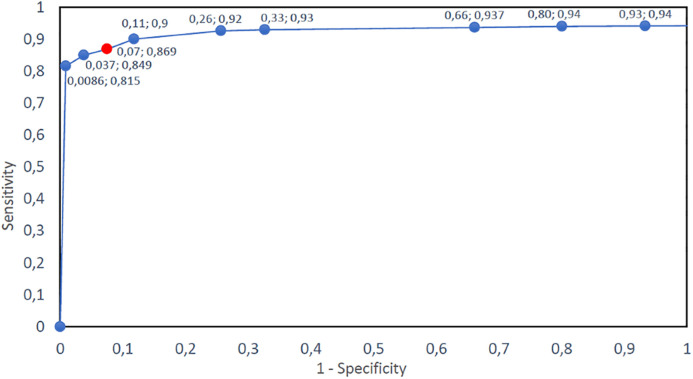
ROC curve corresponding to the evaluation of sensitivity and specificity for different minimum colony sizes.

**Fig 15:**
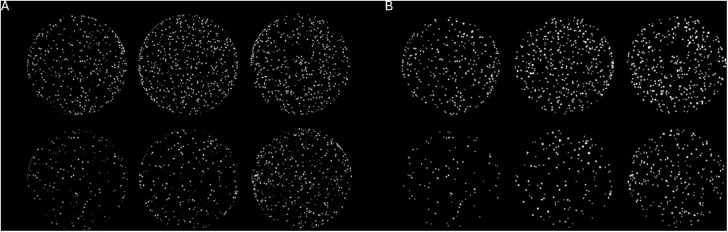
Area-based Filtering for Eliminating Particles Smaller than Eight Pixels in Size. A. Input Image. B. Filtered Image.

To ensure accurate colony counting and avoid the inclusion of cell debris or smaller artifacts that might be inaccurately counted as colonies, it was imperative to establish the minimum colony size. In pursuit of this goal, the minimum acceptable size of each colony was fine-tuned by considering nine possible area values ranging from 2 to 10 pixels. An ROC curve was constructed to evaluate the sensitivity and specificity of each value, as depicted in Fig 14. This comprehensive analysis revealed that the optimal minimum size, which struck the best balance between sensitivity and specificity, was eight pixels. Consequently, particles with areas below eight pixels were systematically excluded, as demonstrated in Fig 15.

## 3. Results

To assess the performance of the developed method, the results were compared with those obtained manually by specialists. The evaluation involved calculating the sensitivity, specificity, and diagnostic accuracy measures. When comparing the methods, the outcomes were categorized as true positives (TP), false positives (FP), false negatives (FN), and true negatives (TN). Table 2 presents the results derived from the comparison between the manual count, utilized as the ground truth, and the results obtained using the developed plugin, referred to as ColCounter, in the six wells of the 17 images.

**Table 2:** Confusion matrix between manual counting and automatic counting method.

		Manual counting		
		Positive	Negative	Total
ColCounter	Positive	7308 (TP)	83 (FP)	7391
	Negative	560 (FN)	1042 (TN)	1602
	Total	7868	1125	8993

Entries represent true positives (TP), true negatives (TN), false positives (FP), and false negatives (FN) obtained when comparing automated detection with manual enumeration as the reference standard.

To determine specificity and sensitivity, the colonies identified in the ground truth images were compared with those detected by the plugin. True positives (TP) were defined as colonies marked in the ground truth that were correctly identified in the plugin-generated images. True negatives (TN) corresponded to background areas or non-colony particles correctly identified as such by the plugin. False positives (FP) were regions incorrectly identified as colonies by the plugin but not present in the ground truth. False negatives (FN) were colonies present in the ground truth that the plugin failed to detect. Sensitivity was calculated as TP/(TP + FN), reflecting the method’s ability to correctly identify actual colonies, while specificity was calculated as TN/(TN + FP), reflecting its ability to correctly reject non-colony objects. It is important to note that the sensitivity and specificity of the automated detection are determined by the algorithm’s ability to identify colony structures in the image rather than by the initial number of seeded cells. Although the number of cells may influence colony density in clonogenic assays, the detection criteria used by the ColCounter plugin are independent of the initial cell count, provided that colonies remain sufficiently separated to allow individual identification.

ColCounter achieved a sensitivity of 92.88% and a specificity of 92.62%, exceeding the values obtained with the same samples and reported by Roldán et al. in 2019, which were 82.3% for sensitivity and 76% for specificity [14]. Roldán et al. also compared their method with the CellCounter and OpenCFU [28] programs, which ColCounter outperformed. The diagnostic accuracy was calculated to be 92.85%, indicating an excellent proportion of correctly identified colonies and non-colony elements using the proposed method. To further enhance the counting process, results can be easily and quickly edited using ImageJ tools, which are integrated into the ColCounter menu upon startup.

Additionally, Table 3 presents the results of the average time required for each well and for all six wells in both the manual counting and ColCounter methods, demonstrating that the proposed method is, on average, more than 10 times faster than manual counting.

**Table 3:** Comparison of manual and automated counting time.

	Manual counting		Colony count	
Images	One well	All wells	One well	All wells
17	72.87 s	437.27 s	6.68 s	40.1 s

Time required to perform colony counting using manual enumeration and the proposed automated method.

In some cases, obtaining images at the resolution specified in the protocol may not always be possible. Therefore, to assess how the developed method performs with images at resolutions different from the ideal, the results obtained from 23 wells scanned at 200 dpi, 60 wells at 300 dpi, and 25 wells at 600 dpi were examined. The overall intraclass correlation coefficient (ICC) was 0.89 (95% CI 0.81–0.93). However, there were variations in the ICC values based on the image resolution. For images scanned at 200 dpi, the ICC was 0.55 (95% CI 0.063–0.808). In contrast, for images acquired at a resolution of 300 dpi, the ICC was 0.96 (95% CI 0.931–0.976). Meanwhile, images scanned at the protocol-specified resolution of 600 dpi exhibit an ICC of 0.99 (95% CI 0.989–0.998).

As expected, the Bland-Altman analysis revealed variations in the limits of agreement depending on the acquisition resolution. Table 4 presents the bias and limits of agreement, along with their 95% confidence intervals, between manual and plugin counts for images acquired at 200, 300 and 600 dpi. For images with a resolution of 300 dpi, the mean difference between methods was 2.96 colonies (0.26 to 5.6), whereas for images at 600 dpi, the mean difference was 4.72 colonies (−5.89 to 15.33).

**Table 4:** Bias and limits of agreement between manual and automated counting.

		Bland-Altman Statistics			
Number of wells	Resolution	Bias (CI 95%)	Upper limit of agreement (CI 95%)	Lower limit of agreement (CI 95%)	p-value^a^
23	200 dpi	106.65 (51.32 to 161.97)	357.40 (261.45 to 453.35)	−144.09 (−240.04 to −48.14)	0.000606
60	300 dpi	2.96 (0.26 to 5.6)	23.46 (18.82 to 28.10)	−17.53 (−22.17 to −12.88)	0.03195
25	600 dpi	4.72 (−5.89 to 15.33)	55.15 (36.73 to 73.51)	−45.68 (−64.07 to –27.29)	0.3679

^a^Alternative hypothesis: The true bias differs from 0.

Bias and limits of agreement between manual and plugin counting for images acquired at 200, 300 and 600 dpi with 95% confidence intervals.

Furthermore, as expected, the limits of agreement between the methods varied for different resolutions. Images at 300 dpi exhibited limits of agreement ranging from 23.46 colonies (18.82 to 28.10) to −17.53 colonies (−22.17 to −12.88). In contrast, images at 600 dpi showed limits of agreement spanning from 55.15 colonies (36.73 to 73.51) to −45.68 colonies (−64.07 to −27.29). It is essential to note that these limits were determined based on the assumption that the difference between methods follows a normal distribution. However, the data for images at 300 dpi did not satisfy this assumption. In contrast, differences in images at 600 dpi appeared to be approximately normally distributed (S1 Fig).

**Fig 16:**
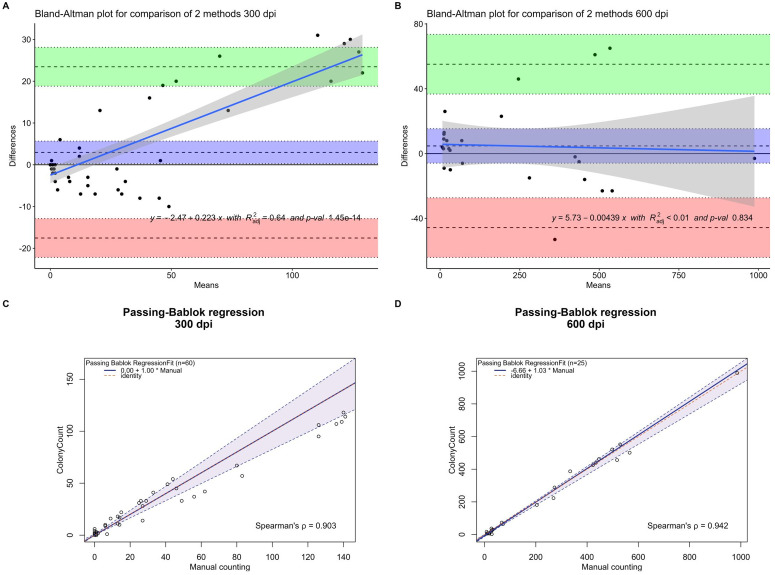
Comparison of results obtained with manual counting and using the ColCounter plugin. A. Bland-Altman plot for the comparison of both methods using 300 dpi images. B. Bland-Altman plot for the comparison of both methods using 600 dpi images. C. Passing-Bablok regression at 300 dpi. D. Passing-Bablok regression at 600 dpi.

To delve deeper into this skewness, further exploration revealed a significant linear relationship for images acquired at 300 dpi, indicating that the difference between methods slightly increased as the number of colonies increased (Fig 16 A). In contrast, no significant trend was observed for images acquired at 600 dpi resolution (Fig 16 B).

Otherwise, a comparison of the methods using the Passing-Bablok regression is presented in Table 5. For images taken at a resolution of 300 dpi, the intercept and regression crossed the null value (0 and 1, respectively). Consequently, there were no constant or proportional differences between manual count and the one performed using the ColCounter plugin (Fig 16C).

**Table 5:** Passing–Bablok regression analysis for automated and manual counting.

Passing-Bablok regression					
600 dpi			300 dpi		
Method	Beta	95% CI	Method	Beta	95% CI
(Intercept)	−6.66	−12.88, −2.41	(Intercept)	0	0.00, 0.51
ColCounter	1.03	0.97, 1.06	ColCounter	1	0.83,1.13

Passing–Bablok regression analysis comparing manual counting and the ColCounter plugin for images acquired at 300 and 600 dpi.

In contrast, for images at a resolution of 600 dpi, the significant intercept revealed a consistent difference between manual counting and that conducted by ColCounter, amounting to −6.66 (95% CI −12.88; −2.41). However, since the intercept crossed the null value of 1, this difference appears to be non-proportional, as it did not escalate with the number of colonies (Fig 16D).

## 4. Discussion

In this study, an automatic method for counting tumor cell colonies was developed and implemented as an ImageJ plugin. The proposed method exhibits high reliability, which surpasses previous applications in accuracy. Because obtaining images with the desired resolution is not always feasible, an investigation into the performance of the proposed method for low-resolution images was conducted. The results revealed that the method maintains a high level of reliability, although it is somewhat dependent on image resolution. For images with a resolution of 300 dpi, the intraclass correlation coefficient (ICC) was 0.96 (95% CI 0.931–0.976), which further improved to 0.99 (95% CI 0.989–0.998) for images with a resolution of 600 dpi.

The analysis of mean bias and limits of agreement between manual and automated counting methods is presented in Table 4. The findings indicated a slight overestimation, with a mean difference of 2.96 colonies (0.26 to 5.6) in automated counting compared to manual counting for images at 300 dpi resolution. For images at 600 dpi resolution, the mean difference was 4.72 (−5.89 to 15.33). Although it exceeded the null value of 0, the confidence interval for this difference was relatively wide.

Additionally, the analysis of the limits of agreement between methods revealed that for images at 300 dpi, 95% of the observations could have differences ranging from 23.46 colonies (18.82 to 28.10) to −17.53 colonies (−22.17 to −12.88). These differences tended to exhibit a positive skew as the number of colonies increased, as indicated in Fig 16. For images acquired at 600 dpi, the limits of agreement were wider. However, in this case, the difference appeared to be independent of the number of colonies.

Furthermore, Passing-Bablok regression analysis demonstrated that there were neither constant differences (Intercept = 0) nor proportional differences (Beta = 1) between the methods when using images at 300 dpi. Conversely, for images at 600 dpi, a constant difference of −6.66 colonies (−12.88, −2.41) was observed between manual and automated counting. Therefore, in this scenario, the plugin consistently underestimated the number of colonies.

No acceptable bias range was pre-established in this study. However, an acceptable method should not systematically over or underestimate the colony counts. The Bland Altman plot statistics tested the alternative hypothesis of a true bias different from zero, thus the rejection of the null hypothesis, suggested a systematic bias with images at 200 dpi and 300 dpi, but no bias with images took a 600 dpi (p = 0.3679). Buryska et al. developed a semiautomated algorithm technique and compared it to microscopic manual counting performed by independent observers [9]. The authors reported a strong correlation between both methods (Pearson correlation coefficient = 0.985 and 0.965 for two observers), with limits of agreement (−50 to +60), and no significant magnitude-dependent bias when comparing the algorithm with microscopic counts, indicating good agreement between the semiautomated and manual microscopic quantification methods [9]. The limits of agreement observed in our study were narrower (±40) than those reported by Buryska et al. Overall, these findings suggest that a semiautomated technique for colony counting can provide reliable results in comparison to traditional methods.

On the other hand, Siragusa et al. [11] identified several critical issues in methods relying on image analysis for clonogenic assays. These concerns encompassed challenges like the need for uniform lighting during imaging, effective background identification, separating fused colonies, developing a rapid calibration procedure, and creating user-friendly and cost-effective methods. Various automated colony counting methods have been proposed in the past 20 years to mitigate the subjective and time-consuming aspects of manual counting. One of the first methods “Clono-counter”, developed in Java [29], based on densitometric and specific image parameters cannot correct for variations in illumination commonly encountered in clonogenic assays. Furthermore, it may not offer user tunability, which limits its adaptability to specific experimental conditions. Another example is the method developed by Guzman et al. [13] called “ColonyArea”, an ImageJ plugin. This approach employs local thresholding by analyzing the first and second derivatives of the percentage area function. Then, it estimates the proportion of surface area covered by viable colonies. Although the tool provides an easy-to-use interface and is freely available, its validation was performed against an alternative method. In addition, the requirement for high-quality images (greater than 800 dpi) may limit its broader applicability

Alternative solutions emerged, such as the one proposed by Choudhry [12], who developed another ImageJ macro called Cell Colony Edge (IMJ Edge) that demonstrated accurate cell and colony detection under various testing conditions, including challenging scenarios like low-contrast images, uneven illumination, and irregularly shaped cells. The IMJ Edge was compared to two alternative digital methods, including the one described in this paper, which showed high correlation with manual measurements. However, IMJ Edge does have some limitations. The method relies on a substantial number of calibration parameters to automate image counting, making it potentially complex to set up and fine-tune. Additionally, it is susceptible to counting issues at the edges of plates and may miscount fused colonies.

Later, the “CoCoNUT” colony counter was developed by the Nutech department at DTU and tested using the V79 and HeLa cell lines [11]. This automated approach is also based on ImageJ and structured as a macro. It relies primarily on a single parameter, specifically, the radius of the smallest counted colony, which contributes to its strong edge detection ability. However, it is worth noting that the requirement for a photographic light box, while cost-effective, could introduce challenges and variations during the image acquisition process.

Interestingly, the development of softwares for the analysis of clonogenic assays has evolved, focusing not only on colony counts but also on other parameters. For example, Militello et al. (2017) and Militello et al. (2020) developed programs that took into account the area covered by colonies to calculate the survival fraction, as the evaluated treatment may not have an effect on the number of colonies, but on their size [15,16]. Similarly, the softwares countPHICS and Schiefer Counter systematically analyze colony size, however, not taking into account its proportionality with the number of colonies to calculate the survival fraction, but as a complement to the statistical analysis of the assay [30,31].

Recent alternatives, such as the ones developed by Gomes et al. and Qian et al., are based on nuclei counting using fluorescence techniques rather than colony size, considering that different treatments may have effects on colony morphology [32,33]. However, the methods are proposed for high-throughput screening. On the other hand, a promising strategy based on the combination of timelapse microscopy, deep learning, and microfluidics has been developed to analyze colony formation [17], nevertheless, the model was applied to cells cultured in 3D systems, which may restrict its application.

In summary, current colony counting methods are often tailored to specific conditions related to image acquisition, lighting, cell morphology, contrast, and uniformity. While progress has been made in leveraging computer technology to address these specific challenges, further developments are needed to create more versatile solutions that can accommodate a broader range of cell lines.

The evaluation of the proposed ColCounter plugin across different image acquisition resolutions highlights several key advantages. In this study, the plugin was tested using images scanned at 200, 300, and 600 dpi to assess its performance under varying acquisition conditions. These advantages include affordability, user-friendliness, and the ability to generate high-quality results. This study significantly contributes to the development of standardized cell and colony counting techniques for clonogenic assays. It is worth noting that the use of a scanner for image acquisition, which is a widely available tool, enhances the method’s practicality and accessibility.

The development of the ColCounter plugin showed low cost, ease of use, and quality results regarding the two cell lines, which helped in the development of a standardized method of cell and colony counting for clonogenic assays. A scanner was used as a standardized image acquisition method that could be widely available. The proposed method showed excellent and interchangeable agreement with manual counting using images at 300 dpi. However, the method also showed some limitations; its performance depended on the resolution of the image acquired, and the colonies at the edges of the plate remained difficult to identify by automated processing. Nevertheless, the plugin included easy-to-use macros that allow users to select and delete colonies for repeat counting, which increases confidence in the method as it can be easily edited by experts.

## 5. Conclusions

Assessing the clonogenic capacity of cells retained after exposure to cytotoxic agents is a valuable tool for evaluating in vitro responses to various therapies. This assay is essential for exploring how cells respond to different cytotoxic agents. The development of automated image processing workflows is of paramount importance as it significantly reduces interobserver variability and enhances the reliability of clonogenic assay studies. Furthermore, automation streamlines the process, thereby reducing the learning curve. In our study, we introduced a novel technique for automatic colony counting that we successfully integrated as an add-on to ImageJ software, achieving a sensitivity of 92.88% and specificity of 92.62%.

Clonogenic assays were used to assess the impact of radiation on a colorectal cancer cell line, comparing manual and automatic counting methods. Future work will focus on optimizing the tool performance for various cell lines and colony morphologies. The study of this specific cell line is particularly significant due to its clinical importance, ranking among the top three worldwide in terms of both incidence and mortality. The newly developed plugin serves as an automated counting method and includes manual editing capabilities, making it a substantial technical contribution to radiobiology research and the work of radiation oncology staff. This system offers an efficient, fast, and accurate approach to quantify tumor cell colonies during clonogenic assays, ultimately advancing cancer radiotherapy.

## Supporting information

S1 Fig Distribution of differences between manual and automated counting. Histograms of differences between manual and automated counting methods for images acquired at 300 dpi and 600 dpi.
(TIF)
